# Polymeric Foams

**DOI:** 10.3390/polym11071179

**Published:** 2019-07-12

**Authors:** Marcelo Antunes, José Ignacio Velasco

**Affiliations:** Departament de Ciència dels Materials i Enginyeria Metal·lúrgica, Universitat Politècnica de Catalunya (UPC·Barcelona Tech), C/Colom 11, E-08222 Terrassa, Barcelona, Spain

Advances in nanotechnology have boosted the development of more efficient materials, with emerging sectors (electronics, energy, aerospace, among others) demanding novel materials to fulfill the complex technical requirements of their products. This is the case of polymeric foams, which may display good structural properties alongside functional characteristics through complex composition and (micro)structure, in which a gaseous phase is combined with rigid ones, mainly based on nanoparticles, dispersed throughout the polymer matrix.

In the last years there has been an important impulse in the development of nanocomposite foams, extending the concept of nanocomposites to the field of cellular materials. This, alongside the developments in new advanced foaming technologies, which have allowed the generation of new foams with micro, sub-micro, and even nanocellular structures, has extended the applications of more traditional foams in terms of weight reduction, damping, and thermal and/or acoustic insulation to novel possibilities, such as electromagnetic interference (EMI) shielding.

This special issue, which consists of a total of 22 articles, including one review article, written by research groups of experts in the field, considers recent research on novel polymer-based foams in all their aspects: design, composition, processing and fabrication, microstructure, characterization and analysis, applications and service behavior, recycling and reuse, etc.

To begin with, an important number of the research articles presented in this issue deal with thermoset-based foams, owing to the fact that polyurethane (PU) foams are still the most important polymeric foams in terms of production and consumption. Chen and co-workers [1] have considered the improvement of one of the most important characteristics of PU foams for vehicle interior purposes: acoustic performance. Namely, they have focused their work on the application of the grey relational analysis (GRA) method and the multi-objective particle swarm optimization (MOPSO) algorithm towards improving the acoustic performance of PU foam composites prepared with ethylene propylene diene monomer (EPDM), setting as optimization objectives the average sound absorption coefficient and the average transmission loss, and as design variables, compositional aspects of the foams such as the content of modified isocyanate. The main conclusion of the authors was that the main factor influencing the acoustic performance of PU foams is the content of added EPDM, with its hardness having the least influence, with the optimum formulation of PU-EPDM foams being obtained by the MOPSO algorithm.

Ma et al. [2] have focused on both experimental and theoretical methods to assess the mechanical and flame spreading characteristics of rigid PU foams used as insulation materials in building façades, clarifying some aspects of downward flame spreading over PU foams, with direct implications in the design of safe vertical façades for high-rise buildings.

Linul and co-workers [3] have addressed the possibility of enhancing the compressive mechanical performance of semi-rigid PU foams by considering the addition of variable amounts of aluminum microfibers (AMs) coming from aluminum solid wastes. Up to an AMs amount of 1.5 wt %, the compressive strength and energy absorption of PU foams increased with incrementing AMs content, reaching improvements of 62% and 71%, respectively, compared to unreinforced PU foams.

Although not taking such a practical and specific approach as Ma and co-workers [2] in their PU-based façade construction, Chen et al. [4] have analyzed the possibility of enhancing the thermal stability and flame retardancy of rigid PU foams using functionalized graphene oxide (fGO) in combination with the well-known intumescent system formed by expandable graphite (EG) and dimethyl methyl phosphonate (DMMP), experimentally demonstrating the improvement of the thermal stability and a decrease in the flammability of PU foams even by adding extremely low amounts of fGO, which acted to strengthen the intumescent char layer formed during burning. In a similar way, Xi and co-workers [5] have used ternary flame retardant systems formed by aluminum hydroxide (ATH), EG and [bis(2-hydroxyethyl)amino]-methyl-phosphonic acid dimethyl ester (BH) to enhance the flame retardancy of rigid PU foams. Interestingly, the authors have demonstrated the synergistic effect between these three components, the combination of the three increasing the limiting oxygen index value, significantly reducing the peak value of the heat release rate, decreasing the mass loss rate, and even inhibiting smoke release during burning.

Owing to the high interest behind the reuse of polymeric foams, especially those that generate a great amount of waste such as PU foams, Gómez-Rojo et al. [6] have considered the possible reuse of PU foam waste coming from the refrigeration and automotive sectors for building materials, focusing on some of the required characteristics of said materials, namely mechanical performance, thermal stability, and fire retardancy. This work comes as an initial study required to determine the main characteristics and differences between PU foam residues coming from different industries and hence their possible reuse for building materials.

Greatly expanded among the scientific community is the possibility of adjusting the properties of foams by means of adding different types of (nano)reinforcements, and more recently the development of almost tailor-made (nano)reinforcements for specific types of foams. In this sense, Ma and co-workers [7] have considered the preparation and characterization of modified ethyl cellulose (EC) for enhancing the mechanical performance and fire retardancy of phenolic foams. Specifically, the authors directly attached 9,10-dihydro-9-oxa-10-phosphaphenanthrene-10-oxide (DOPO) and itaconic acid (ITA) to the backbone of ethyl cellulose and added the modified EC to phenolic foams, with the resulting foams displaying enhanced mechanical performance and improved flame retardancy.

Much in the same way as a great deal of other research groups, which have considered the design of polymer foams with improved mechanical properties by adding secondary phases, trying to counteract the inherent loss in mechanical performance after foaming, Back and co-workers [8] have analyzed the possibility of enhancing the resistance to cleavage of epoxy-based structural foam adhesives for automotive applications by adding core shell rubber (CSR) particles. Through a set of experimental tests, the authors demonstrated that at relatively low amounts of foaming agent and 20 phr of CSR it was possible to maximize the impact resistance of the foam adhesive while keeping the required expansion ratio, extending its use as high performance light structural adhesive.

Thermoset-based syntactic foams, especially phenolic- and epoxy-based, have a vast use with the addition of hollow glass microspheres. Nevertheless, there is a high probability of rupture of these glass microspheres during processing, hence the interest in replacing them with expandable polymeric microspheres. In this sense, Martín-Gallego et al. [9] have considered the development of epoxy-based foams using expandable polymeric microspheres, adding low amounts of carbon-based nanoparticles for enhancing the transport properties, namely thermal and electrical conductivities. The authors have demonstrated the viability of developing epoxy-based foams with considerable weight reductions and electrical conductivities that were five orders of magnitude higher than the reference material at only 0.5 wt % of carbon nanotubes, while the thermal conductivity increased up to 20%, demonstrating the possibilities of these foams as lightweight cores in sandwich-like structures or as EMI shielding elements in electronic components.

Albeit less considered than melt-foaming, especially at the industrial level, solution foaming is still a frequently used foaming technique, owing to some obvious advantages such as lower processing temperatures and easier addition of secondary phases. The work presented by Wang and co-workers [10] has considered the preparation of polyamide 6 (PA6) foams by previously dissolving PA6 and mixing sodium carbonate with formic acid, thus obtaining foams with hierarchical porous structures with cells oriented along the foaming direction and sizes that ranged from hundreds of nanometers to several micrometers, depending on the amount of sodium carbonate/formic acid. As a consequence, PA6-based foams with variable mechanical performances and good thermal insulation could be developed, opening up their use for engineering applications. 

As a result of the generation and entrapment of a well-defined gas phase and cell formation, foaming tends to result in a decrease in some mechanical characteristics of the material, such as tensile or compressive strength, especially in the case of thermoplastic-based foams, where almost all available industrial foaming methods are based on melt-foaming processing techniques. As a consequence, a great deal of research efforts have been focused on counteracting these mechanical losses, mainly by adding secondary phases that promote heterogeneous cell nucleation and favor the formation of finer cellular structures with enhanced mechanical performance at equal density, or that in some cases help to adjust the crystallinity of the polymer matrix, or secondary rigid phases that act as mechanical reinforcement. This special issue includes works that deal directly with the mechanical improvement of polymer foams using said strategies, as is the case of Gong et al. [11], Yang et al. [12], and Wang et al. [13] articles. In all of these, the addition of secondary phases—in the case of the first two, elastomeric in nature, and in the case of the third, a natural nanofiber—promoted the formation of polypropylene (PP) foams having smaller cell sizes and higher cell densities by means of heterogeneous cell nucleation, ultimately resulting in PP-based foams with improved mechanical performance. Particularly, Gong et al. [11] have shown how the presence of an elastomeric phase led to PP foams with much better impact performance at extremely low temperatures, while at higher temperatures the higher impact strength was related to the finer cellular structure of the filled foams. Yang et al. [12] and Wang et al. [13] have presented similar results for tensile and compressive strengths, with PP foams containing the elastomeric phase in the case of the first, and cellulose nanofibers in the case of the second, displaying significantly higher mechanical performance when compared to their unfilled counterparts. In the same way, Aksit et al. [14] have shown how the addition of small amounts of a foam nucleating agent led to extruded polystyrene (XPS) foams with enhanced compression modulus by effectively favoring the formation of finer and more homogeneous cellular structures while keeping the density low, the combination of which resulting in foams with lower thermal conductivity with obvious applications as structural thermal insulating components. Fei and co-workers [15] have considered the addition of lignin or carbon nanotubes/micrographite to XPS foams in order to control the cellular morphology of the extruded foams, and as a consequence, adjust their mechanical performance and acoustic absorption properties. Although the addition of small amounts of carbon nanotubes/micrographite clearly led to improvements in the compressive strength due to the formation of foams having finer cellular structures, the addition of high amounts of lignin resulted in a decrease in compressive strength, which was related to a separation of lignin from the PS matrix. Though displaying poorer mechanical properties, XPS composite foams containing lignin displayed better acoustic absorption properties, showing promising characteristics for sound absorption applications. Realinho et al. [16] have developed injection-molded ABS-based microcellular foams with improved fire retardancy and enhanced storage modulus by adding a combination of halogen-free flame retardants, which favored the formation of integral foams having a microcellular core with smaller cell sizes and higher cell densities, resulting in foamed components with improved mechanical performance.

One of the most popular topics of polymer composite foams considers the possibility of taking advantage of their cellular structure and the addition of electrically conductive secondary phases (besides additional magnetic third phases) to create novel lightweight components with enhanced EMI shielding. In this special issue, a study by Li et al. [17] has focused on preparing cellulose-based foams with improved EMI shielding by adding short or long carbon fibers. The authors demonstrated the efficient formation of conductive networks by using both types of fibers during foaming, resulting in specific EMI shielding effectiveness values from 10 to 60 dB. In the same way, Yan and co-workers [18] have considered the development of polymethacrylimide (PMI) foams with improved EMI shielding performance, focusing on EMI absorption, by adding electromagnetic absorbers during foaming, and combining said foam with metallic tubes to guarantee proper compressive performance, thereby creating a component that could find interesting applications in electromagnetic wave stealth load-carrying structural applications. Abbasi and co-workers [19] have developed microcellular polyetherimide (PEI) nanocomposite foams containing low amounts of graphene nanoplatelets, being able to reach high electrical conductivities while significantly reducing density, which could enable their use in cutting-edge sectors such as aerospace or telecommunications for applications requiring electrostatic discharge (ESD) or EMI shielding. Petrossian et al. [20] have taken this strategy and created thermoplastic polyurethane (TPU)-lead zirconate titanate (PZT) piezocomposite foams with lower electrical permittivity and improved voltage sensitivity, as foaming favored the dispersion of PZT agglomerates during foaming, broadening the applications of piezocomposites while reducing the minimum required amount of PZT particles.

Mansour et al. [21] have considered the interesting subject of polymer/surfactant layer interactions, particularly segregation vs. interdigitation, as a way to understand the mechanisms behind the dynamic interfaces between a solid continuous matrix and gas phase(s) in a polymer foam system. Particularly, they have explored the nanoscale structures present at dynamic interfaces in the form of air-in-water foams, demonstrating that weak solution interactions led to segregation at the foam interface while strong interactions led to interdigitated layers, driving the characteristics of the final foamed system.

To finish this special issue dedicated to polymeric foams, an interesting review of the shock-driven decomposition of polymer foams is presented by Dattelbaum and Coe [22], with special importance being given to the application of high-level state equations to analyze the decomposition products of polymer foams under shock loadings.
